# A new species of *Caligus* (Copepoda, Siphonostomatoida) from the plankton of the Caribbean coast of Venezuela with a key to species

**DOI:** 10.3897/zookeys.201.3099

**Published:** 2012-06-14

**Authors:** Eduardo Suárez-Morales, Humberto Camisotti, Alberto Martín

**Affiliations:** 1 El Colegio de la Frontera Sur (ECOSUR), Av. Centenario Km. 5.5, A.P. 424, Chetumal, Quintana Roo 77000, Mexico; 2 Laboratorio de Crustáceos Peracáridos, Departamento de Estudios Ambientales e INTECMAR, Centro de Biodiversidad Marina. Universidad Simón Bolívar, A.P. 89000 Caracas, Venezuela

**Keywords:** Bahía Amuay, marine zooplankton, crustaceans, biodiversity

## Abstract

During a survey of the zooplankton community of Bahía Amuay, Venezuelan Caribbean, specimens of an undescribed species of *Caligus* Müller were collected. It resembles *Caligus xystercus* Cressey and *Caligus ocyurus* Cressey, both known only from the Caribbean Sea. The new species can be distinguished from these and other congeners by a combination of characters including the armature of legs 1 and 4, but mainly by its unique female genital complex. This is the first species of *Caligus* described from Venezuela. The species is described in full and a key to the species of the genus recorded in Venezuela is provided.

## Introduction

The siphonostomatoid copepods of the genus *Caligus* Müller, 1785 are one of the most diverse and representative crustacean parasites of teleost fish (Boxshall and Halsey 2004; Ho and Lin 2004). They are usually recorded as ectoparasitic forms attached to the hosts, but in many cases they can be found in the plankton (Venmathi Maran and Ohtsuka 2008). Records of parasitic copepods of Venezuelan marine teleosts are relatively scarce (Luque and Poulin 2007). This is particularly true in the Caribbean Sea, a region in which the parasitological research is lagging despite the high number of parasite species harbored by Caribbean fish (Bunkley-Williams and Williams 1994; Luque and Poulin 2007). Previous surveys of the caligid fauna of Venezuela are relatively scarce and up to 10 species of *Caligus* have been recorded from this country (Ho and Bashirullah 1977; Legarde 1989; Williams and Bunkley-Williams 1996; Díaz Díaz 2000; Zambrano et al. 2003).

A recent (February 21–24, 2012) biological survey of the planktonic fauna was carried out at the Bay of Amuay, a coastal system on the northern coast of Venezuelan Caribbean coast, which is also an important oil extraction site with pollution problems (Pomares Ferraz et al. 2008). Specimens of the copepod genus *Caligus* were recorded in these samples. These individuals represent a new species which is described here and compared with its closest congeners. An identification key to the species of *Caligus* recorded from Venezuela is also provided.

## Material and methods

Adult female and male individuals of a caligid copepod of the genus *Caligus* were recovered from plankton samples obtained from Bahía Norte, a shallow coastal system which is part of the Bay of Amuay (11°46'32"N, 70°13'51"W), in the northwestern coast of Venezuela. The plankton fauna from this area was surveyed during several days (February 21–24, 2011) by performing horizontal hauls with a standard plankton net (0.5 mm mesh size, 0.3 m mouth diameter). Specimens were fixed shortly after collection in 70% ethanol. Specimens of *Caligus* were sorted from these samples and processed for identification by transferring them to glycerol and then pure glycerine. Drawings were prepared using a camera lucida mounted on an E-200 Nikon compound microscope. Terminology of the body parts and appendages follows Ho and Lin (2004). Type specimens were deposited in the collection of Zooplankton held at El Colegio de la Frontera Sur (ECOSUR), Unidad Chetumal (ECO-CHZ), Quintana Roo, Mexico.

## Systematics

### Order Siphonostomatoida Thorell, 1859. Family Caligidae Burmeister, 1835. Genus *Caligus* Müller, 1785

#### Caligus evelynae sp. n.

urn:lsid:zoobank.org:act:40EC2919-0E77-4150-B670-C74AC8C3CD0A

http://species-id.net/wiki/Caligus_evelynae

Figs 1 [Fig F2] –3

##### Material examined.

Holotype female, collected February 21, 2011 by D. Arocha, D. Querales and F. Cancines, Bay of Amuay, Venezuela. Specimen undissected, ethanol-preserved, vial (ECO-CHZ-07565). Allotype male, same date, collector, and site, undissected, ethanol-preserved, vial (ECO-CHZ-07566).

##### Type locality.

Bay of Amuay, Venezuelan Caribbean, 11°46'32"N, 70°13'51"W, plankton.

##### Host.

Unknown.

##### Description of female.

**Figure 1.**
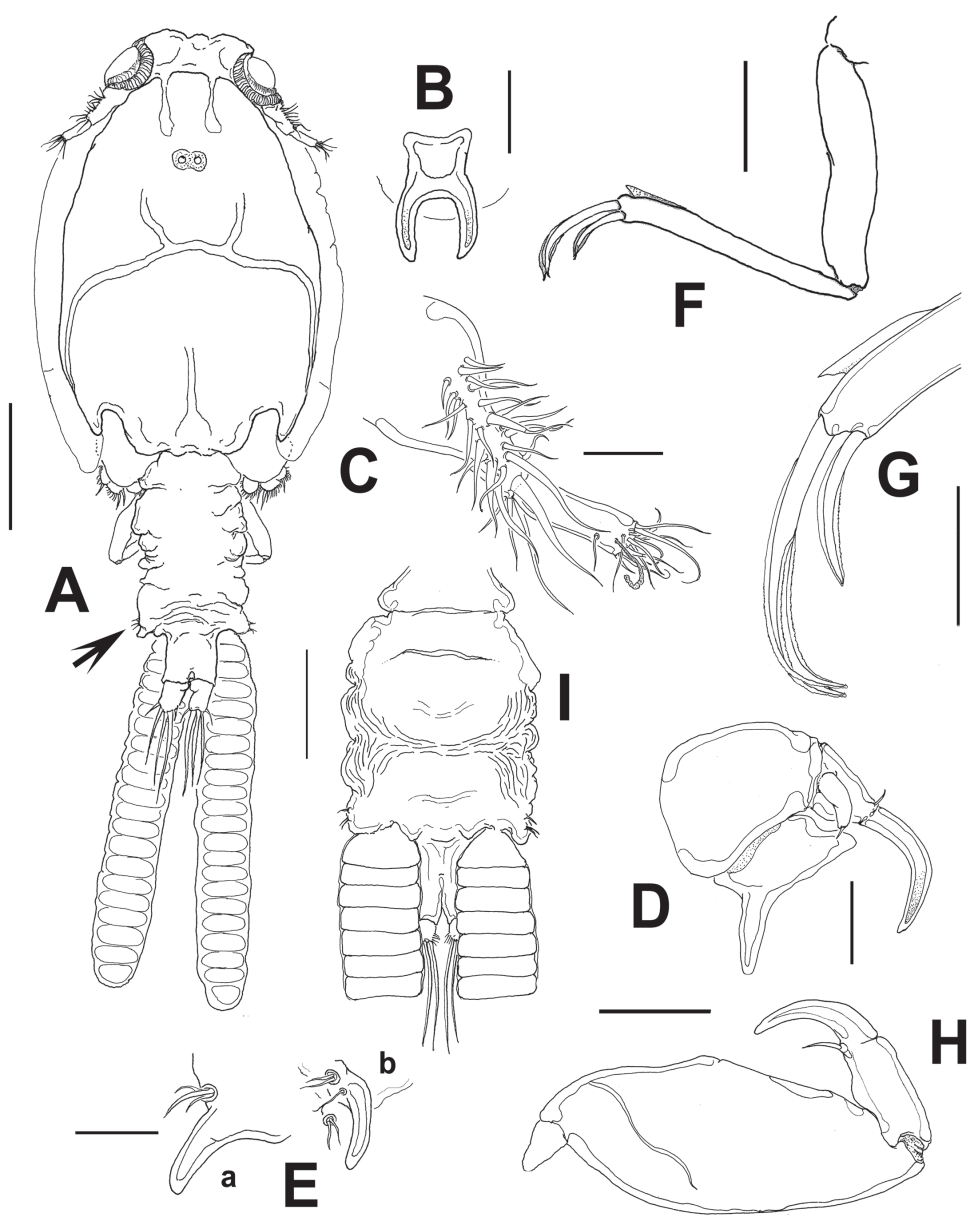
*Caligus evelynae* sp.n., adult female from Venezuela: **A** habitus, dorsal view **B** sternal furca, ventral view **C** antennule **D** antenna **E** postantennal process (**b**) and maxillule (**a**) **F** maxilla **G** detail of calamus and canna **H** maxilliped **I** genital complex and abdomen, ventral view. Scale bars: **A, I**=0.5 mm, **B–F, H** =0.1 mm, **G**=0.05 mm.

**Figure 2. F2:**
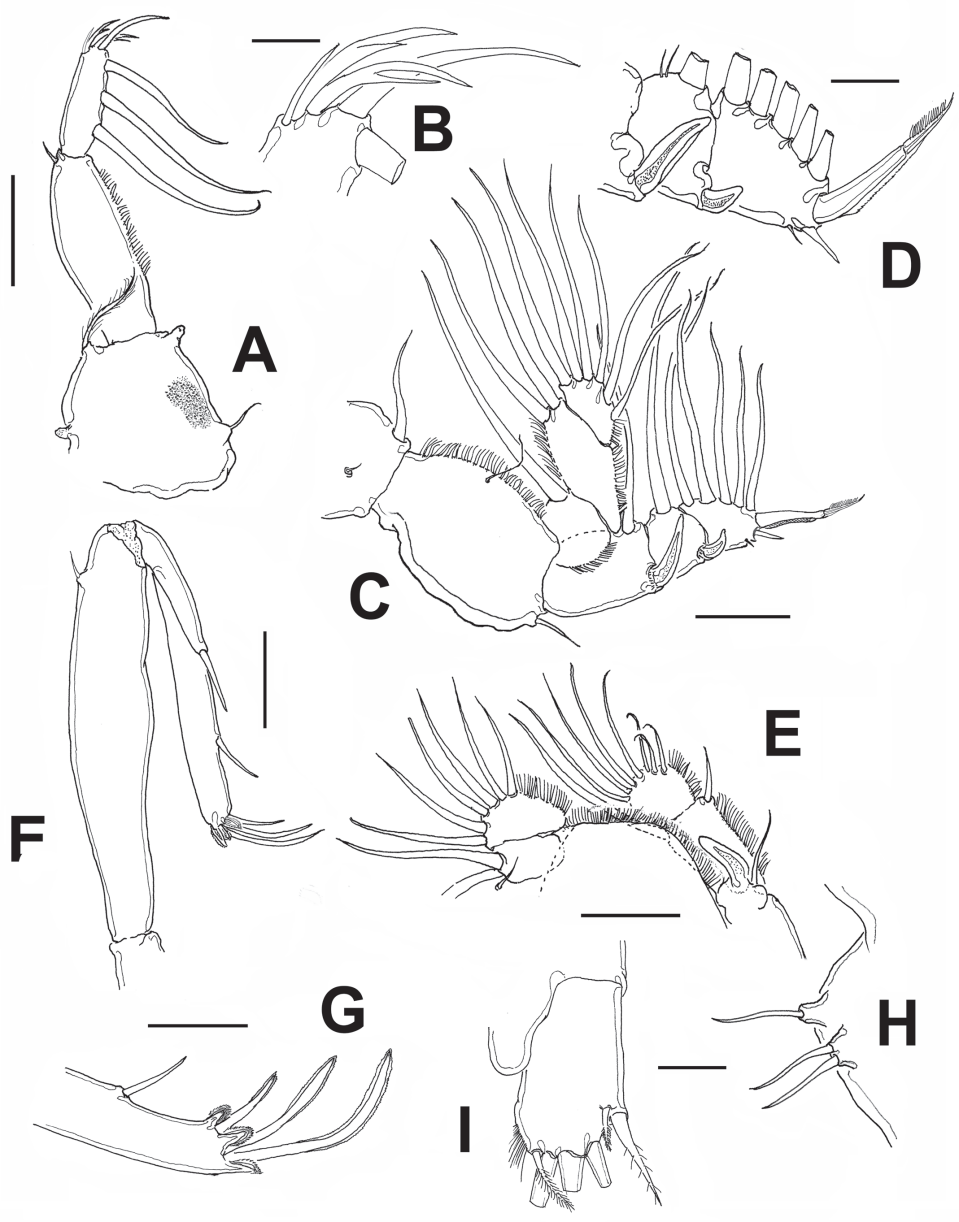
*Caligus evelynae* sp.n., adult female from Venezuela: **A** first leg **B** detail of distal elements of first leg **C** second leg **D** detail of exopodal segments of second leg **E** third leg **F** fourth leg **G** detail of terminal elements of fourth leg **H** fifth leg **I** caudal ramus, dorsal. Scale bars: **A,C, F, E**=0.1 mm, **B, D, G, H, I**=0.025 mm.

Body shape as shown in Fig. 1A, with cephalic shield ellipsoidal with curved lateral margins. Total length 2.55 mm, greatest width 1.1 mm (measured at widest part of cephalothorax). Cephalothorax comprises more than half total length (1.33 mm). Genital complex longer than wide (1.2 x 0.71 mm) with irregularly undulated outer margins and rugged ventral and dorsal surfaces; posterolateral region protruding posteriorly. Abdomen subquadrate, about as long as wide, genital complex approximately 3.4 times longer than abdomen. Caudal rami subrectangular about 1.2 times longer than wide, armed with 3 long terminal, one small outer and one small inner pinnate setae (Fig. 2I). Lunules spaced by the length of about 1.5 times the lunule diameter.

Antennule (Fig. 1C) with the usual structure found in *Caligus*, 2-segmented, proximal segment distinctly longer than distal segment, armed with 22 plumose setae. Distal segment bearing 12 setae (1 of which is subdistal) plus two aesthetascs.

Antenna (Fig. 1D) claw recurved at right angle near tip with small, proximal accessory process; posterior process heavily sclerotized, pointed, but not sharply so. Postantennal process (Fig. 1Eb) sickle-shaped, with rounded tip, with two basal papillae, proximalmost armed with three setae, the other with a single, branched seta. Another papilla with two setae located nearby on sternum.

Maxillule represented by bluntly pointed subtriangular process and basal papilla bearing three small setae (Fig. 1Ea). Maxilla (Figs 1F, G) 2-segmented, brachiform; proximal segment (lacertus) unarmed; distal segment (brachium) slender, with subdistal flabellum on outer margin. Terminal elements, calamus and canna unequally long, the latter about half the length of the former. Maxilliped (Fig. 1H) robust, without protrusions in basal region; subchela about one-half length of basal segment. Terminal claw slightly shorter than shaft, armed with short proximal seta.

Sternal furca (Fig. 1B) tines slightly incurved, membranous on outer margin and longer than base. Leg 1 (Fig. 2A) coxa with patch of very fine spinules in addition to long setulated outer seta and short inner seta. Mammiliform papilla on distal position of segment. First exopodal segment with row of short hair-like elements. Last exopod segment bearing 3 medial pinnate setae and 2 terminal spines, each with accessory process, with additional medial distal seta; usual small seta on outer terminal corner not seen (Fig. 2B).

Leg 2 (Fig. 2C) coxa small, with long plumose seta on inner margin and setule-bearing papilla on middle-outer surface. Basipodite robust, with small seta on outer edge and a setule-bearing papilla on middle inner margin. First exopodal segment bearing spine reaching across second segment to proximal margin of third segment; second segment with much shorter spine recurved on outer margin, spine reaching to middle of last segment. Distal segment with short spine on outer margin plus one short setule. Terminal spine sclerotized, articulated, as long as distal segment (Fig. 2D). All setae on medial margins of all segments pinnate.

Leg 3 (Fig. 2E) exopod first segment with stout, slightly recurved, terminal spine with thin flange on outer lateral margin nearly reaching to third segment; setae as in figure and typical of genus; second endopodal segment with single inner pinnate seta, second segment with 6 pinnate setae.

Leg 4 (Figs 2 F, G) uniramous, brachiform; exopod 2-segmented. Protopod with short plumose seta in distal outer margin. First exopodal segment with terminal seta not reaching base of middle lateral seta of last segment. Second exopodal segment bearing 3 unequally long setae, outermost shortest and medial longest; each with pecten at base.

Armature of rami of legs 1–4 as follows (Roman numerals indicating spines and Arabic numerals, setae):

**Table:** 

exopod	endopod	
Leg 1	1-0; III, 4	vestigial
Leg 2	I-1; I-1; II, 5	0-1; 0-2; 6
Leg 3	I-0; I-1; III, 4	0-1; 6
Leg 4	I-0; I, III	absent

Leg 5 represented by two small papillae on posterolateral corner of genital complex, one armed with two setae, the other with one seta (arrowed in Fig. 1A, Fig. 2H).

##### Description of male.

**Figure 3.**
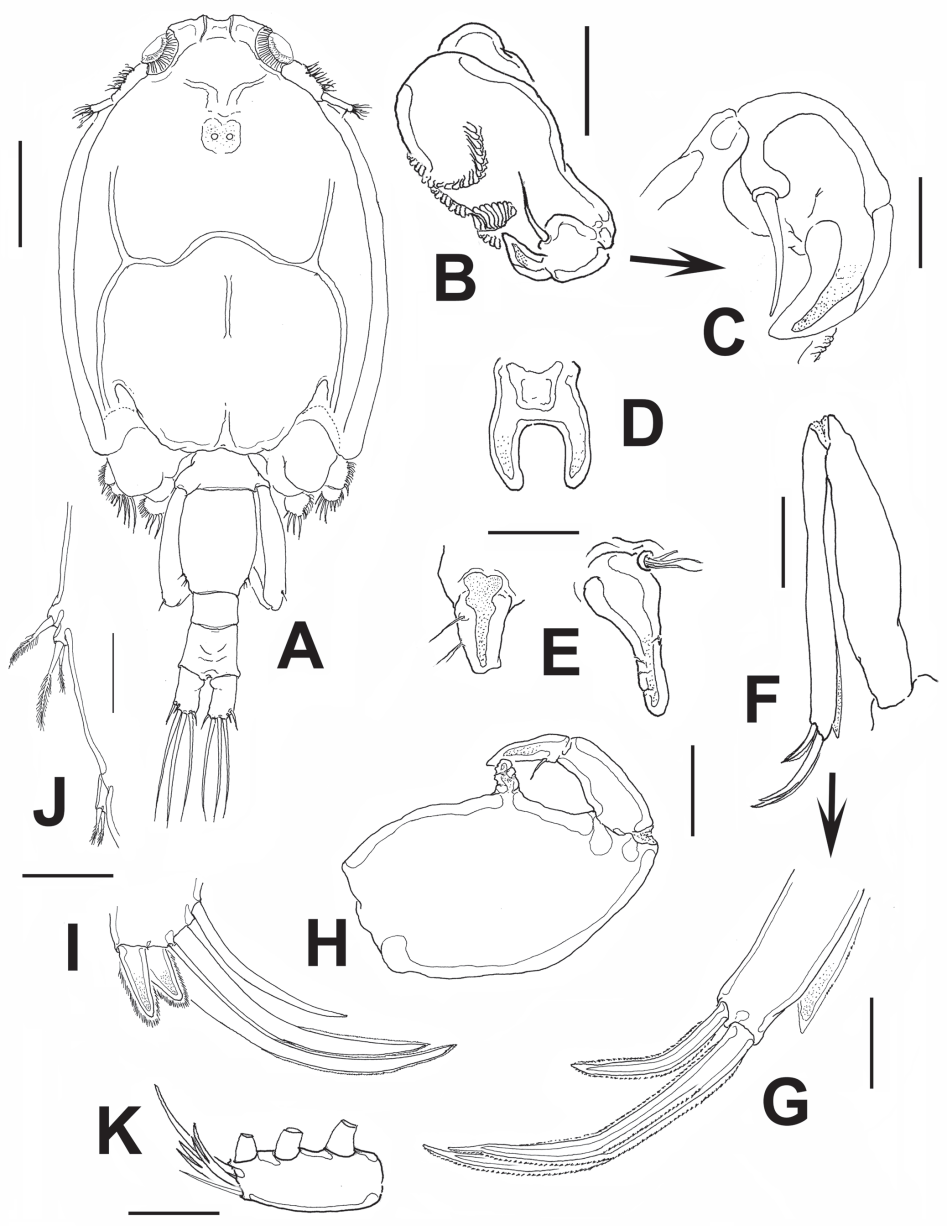
*Caligus evelynae* sp.n., adult male from Venezuela: **A** habitus, dorsal view **B** antenna **C** detail of distal part of antenna **D** sternal furca, ventral view **E** postantennal process and maxillule **F** maxilla **G** detail of calamus and canna **H** maxilliped I fourth leg, detail of distal elements **J** fifth and sixth legs **K** first leg, distal segment of exopod. Scale bars: **A**=0.5 mm, **B,D–F, H**=0.1 mm, **C, G, I, J**=0.03 mm; **K**=0.07 mm.

Body (Fig. 3A) larger than female, 3.1 mm long excluding setae on caudal rami. Cephalothoracic shield roughly ovoid in shape, 1.78 mm long and 1.35 mm wide (excluding marginal hyaline membranes: 0.07 mm). Frontal plates well developed and carrying moderately large lunules separated by 1.5 the diameter of a lunule; free margin of thoracic zone projecting slightly beyond tips of lateral zones; sinuses moderately deep. Fourth pediger separated from genital complex, roughly hexagonal in shape, about twice longer than wide. Genital somite subrectangular. Abdomen 1mm long, represented by two somites; proximal somite subquadrate, anal somite distinctly longer than wide. Caudal ramus subrectangular, slightly longer than wide, bearing 3 short (one inner, two outer) setae, and 3 long terminal setae. Inner margin naked.

Antennule as in female.

Antenna (Fig. 3B, C) 3-segmented; proximal segment slender, unarmed, second segment largest, armed with 3 corrugated pads, 1 along inner margin, 2 transverse on outer surface; terminal segment smallest, armed with single basal seta and short, robust hook.

Sternal furca as in female, tines slightly more robust (Fig. 3D). Postantennal process short, distally truncate, armed with one proximal and two medial setae (Fig. 3E).

Maxillule (Fig. 3E) longer than in female, comprising distally blunt dentiform process bearing basal papilla armed with one short and two long setal elements.

Maxilla (Fig. 3F, G) 2-segmented; proximal segment (lacertus) unarmed, slightly shorter than brachium; distal segment (brachium) with subterminal hyaline membrane and two terminal unequal elements (canna about half as long as calamus).

Maxilliped (Fig. 3H) 3-segmented, robust, first segment with medial cylindrical protuberance; second and third segments forming a subchela with subterminal seta.

Leg 1 (Fig. 3K), 2, 3, and 4 as in female, except for relatively shorter outermost terminal claw (Fig. 3I).

Leg 5 (Fig. 3J) located on middle of lateral margin of genital complex, represented by two papillae, one (proximalmost) armed with short seta and the other with two short, slender setae. Leg 6 (Fig. 3J) represented by single papilla armed with two short, pinnate seta.

##### Etymology.

The new species is named after Dr. Evelyn Zoppi de Roa, an esteemed Venezuelan researcher who pioneered much of the zooplankton research in this country and has lead new generations of planktologists during her long career.

##### Remarks.

This species is closely related to other two species of *Caligus* described from the Northwestern tropical Atlantic: *Caligus ocyurus* Cressey, 1991and *Caligus xystercus* Cressey, 1991, they all share a longer than wide genital complex, a relatively short abdomen, a similar structure and armature of legs 1 with three inner setae on the last segment and four distal elements, two of them with accessory spines. They have also a fourth leg armed with 5 spines; the three terminal spiniform elements are of different lengths, the outermost being longest. Cressey (1991) distinguished *Caligus ocyurus* from *Caligus xystercus* by the proportions of the caudal rami and the abdomen; these structures are not longer than wide in *Caligus xystercus*, as it occurs in *Caligus ocyurus*. Also, the postantennal process of *Caligus ocyurus* is much stouter and broader at the tip than in *Caligus xystercus*. The new species has a postantennal process as in *Caligus xystercus*, not spatulate or particularly wide distally as in *Caligus ocyurus*. In *Caligus xystercus* the caudal rami are equally long as wide and in *Caligus ocyurus* they are twice as long as they are wide, whereas they are 1.5 times longer than wide in *Caligus evelynae*. The new species shares with *Caligus ocyurus* a claw-like terminal segment of the male antenna, a relatively short outermost distal seta of the second exopodal segment of leg 1, and the female is smaller than the male. The new species can be readily distinguished from *Caligus ocyurus* by the shape of the cephalothoracic shield, which is clearly ellipsoidal with curved lateral margins in *Caligus evelynae* sp. n. (see Fig. 1A) and it is rectangular with straight lateral margins in *Caligus ocyurus* (Cressey 1991, fig. 127). In *Caligus evelynae* sp. n., the basipodal segment of leg 1 lacks a large medial protuberance present in *Caligus ocyurus* (Cressey 1991, fig. 131). The sternal furca is clearly different in both species; tines are straight, relatively long, widely separated, and distally tapered in *Caligus ocyurus* (Cressey 1991, fig. 130) whereas they are shorter, more robust, incurved and distally rounded in *Caligus evelynae*. The maxillule is slender in the new species vs. a stronger, more robust condition in *Caligus ocyurus* (Cressey 1991, fig. 129c). The armature of leg 4 is different; distal elements differ in length and proportion in both species; the size of the short outermost element is about 1/3 the length of the medial element, which in turn is about half the length of the innermost element; this pattern differs in *Caligus evelynae* sp. n., in which the shortest element is about half the length of the medial one and this is about 0.75 times as long as the innermost element. In *Caligus ocyurus* the male genital somite is as long as wide, with protuberant lateral margins (Cressey 1991, fig. 135), whereas in the new species this somite is longer than wide with weakly produced lateral margins. The male antenna is described as bifid by Cressey (1991) and it is not bifid in the new species. Also, the size and position of the pads differ in the new species (see Fig. 3B vs Cressey 1991, fig. 136); particularly, the new species has a longitudinal adhesion pad along the inner margin whereas this margin is smooth in *Caligus ocyurus* (Cressey 1991, fig. 136).

Following Cressey’s (1991) criteria, these three species can be separated from the other known *Caligus* species of the Gulf of Mexico and Caribbean Sea by the following characters. The structure and armature of the exopod of the fourth leg with a 2-segmented exopod bearing 5 spines is a character shared by at least 11 species of the genus in this region (Cressey 1991). This group of species can be distinguished from C. *asperimanus* Pearse, 1951,* C. berychis* Wilson, 1935,* C. bonito* Wilson, 1905,* C. haemulonis* Krøyer, 1863,* C. mutabilis* Wilson, 1905, and C. *suffuscus* Wilson, 1913in having short setules along the outer margin of the second leg endopod instead of patches of spinules. This group of species can be distinguished from the remaining 6 other species with setules on the second leg endopodal segments as follows: the female *Caligus chelifer* Wilson, 1905has a 2-segmented abdomen and very short lateral setae on the exopod of leg 1, vs. a 1-segmented female abdomen and relatively longer setae on the leg 1 exopod; *Caligus praetextus* Bere, 1936bears unique “hooded” distal spines on the exopod of leg 1, and two of the terminal elements of leg 4 are equally long, about half the length of the inner element, *Caligus afurcatus* Wilson, 1913has also two equal terminal elements on leg 4, reduced caudal rami, relatively short lateral seta of the distal exopodal segment of leg 1, a reduced sternal furca, and a relatively long outer spine on the first exopodal segment of leg 2; in C. *rufimaculatus* Wilson, 1905the sternal furca tines are clearly spatulate, with an enlarged distal margin, caudal rami only slightly longer than wide, *Caligus productus* Dana, 1852lacks medial lateral setae on leg 1 and has no accessory spines on leg 1 exopod.

Overall, the main character distinguishing the new species, *Caligus evelynae*, is the peculiarly strong, irregular undulation of the genital complex; another species with a genital complex bearing undulated margins reported in the region is *Caligus undulatus* Shen and Li, 1959 (Suárez-Morales et al. in press), but in this species the first leg has a different armature, with four distal elements, the genital complex is trapezoidal vs. a subrectangular shape in the new species; the abdomen is shorter in *Caligus evelynae* (30% length of genital complex), whereas in *Caligus undulatus* the abdomen is clearly longer (67%). Also, undulation in *Caligus undulatus* is relatively shallow and regular, contrasting with the pattern found in *Caligus evelynae*. This species was originally described from specimens in plankton samples from China (Shen and Li 1959) and has been recorded only from the water column, hosts remain unknown (Venmathi Maran and Ohtsuka 2008). Other species of *Caligus* with an undulate or rugose genital complex are *Caligus lobodes* (Wilson, 1911) and *Caligus rugosus* Shiino, 1959. The new species differs from the former in the body shape, which is quite different, the genital complex is trapezoidal and the abdomen is as wide as the genital complex; lobes are symmetrical, lunules are widely separated from each other and it has a forked maxillular process (Wilson 1911), thus differing from the pattern described for *Caligus evelynae*. In *Caligus rugosus* the genital complex is also irregularly lobulated but it is produced posteriorly, forming large posterolateral processes which are absent in the new species. Also, the sternal furca has very wide, wing-like tines (Shiino 1959, fig. 1G) *vs*. regular, slender tines in *Caligus evelynae*.

It is interesting to point out that these three very similar species of *Caligus* (*Caligus xystercus*, *Caligus ocyurus*, *Caligus evelynae*) appear to be restricted to the Northwestern Atlantic (Cressey 1991) following the pattern described by Ho and Lin (2003) in reference to another group of Old World species of *Caligus* with a well-defined geographic affinity. This criterion motivated their revision of New World records of these Old World species and the naming of a new species from the Caribbean Sea, which was formerly identified as *Caligus epinepheli* Yamaguti, 1936 by Cressey (1991). Hence, it appears that this group of related species from the Gulf and the Caribbean basins, in which we include also *Caligus rufimaculatus*, are probably restricted to this region. Based on the available data, there are about 31 nominal species of *Caligus* known in the Gulf of Mexico and the Caribbean (Wilson 1913; Cressey 1991; Bunkley-Williams and Williams 1994; Álvarez-León 2009; Suárez-Morales et al. 1998, 2003, in press). Only a few of them are known to be restricted to this geographic region, including *Caligus xystercus*, *Caligus ocyurus*, *Caligus kabatae* Cressey, 1991, *Caligus pomacentrus* Cressey, 1991, *Caligus cresseyi* Ho and Lin, 2003, and probably the new species, *Caligus evelynae*. More taxonomical surveys are yet to be developed in order to reveal more of the potentially highly diverse caligid fauna in the region.

### Key to the species of *Caligus* recorded from Venezuela (females)

**Table:** 

1	Leg 4 exopod with three segments	2
–	Leg 4 exopod with two segments	4
2	Abdomen more than three times as long as cephalothoracic shield	*Caligus bennetti* Causey, 1953
–	Abdomen about as long as or slightly longer than cephalothoracic shield	3
3	Inner margin of second exopodal segment of leg 1 with three short setal elements, without postero-lateral process on genital complex	*Caligus chorinemi* Krøyer, 1863
–	Inner margin of second exopodal segment of leg 1 unarmed; rounded, strongly developed postero-lateral processes on genital complex	*Caligus productus* Dana, 1852
4	Abdomen one segmented	5
–	Abdomen two or three-segmented	10
5	Abdomen short, about 0.2 times as long as genital complex	*Caligus atromaculatus* Wilson, 1913
–	Abdomen not as short	6
6	Abdomen about 0.6 times as long as genital complex, without postero-lateral processes on genital complex	7
–	Abdomen as long as genital complex (or even longer), with strongly protruding postero-lateral processes on genital complex	*Caligus bonito* Wilson, 1905
7	Inner margin of second endopodal segment of leg 2 armed with short, slender setae	8
–	Inner margin of second endopodal segment of leg 2 armed with tooth-like elements	*Caligus asperimanus* Pearse, 1951
8	Genital complex rugose, with undulate lateral margins	*Caligus evelynae* sp. n.
–	Genital complex differently built	9
9	Middle two of the terminal elements of leg 1 with accessory process	*Caligus rufimaculatus* Wilson, 1905
–	Middle two of the terminal elements of leg 1 without accessory process	*Caligus constrictus* Heller, 1865
10	Abdomen three-segmented	*Caligus coryphaenae* Steenstrup & Lütken, 1861
–	Abdomen two-segmented	11
11	Claws of leg 4 decreasing in length from the inner to outer margin, inner setae of distal exopodal segment of leg armed with spines	*Caligus mutabilis* Wilson, 1905
–	Claws of leg 4 with middle and outer elements being equally long and about half the length of inner claw, inner setae of distal exopodal segment of leg setulated	*Caligus irritans* Heller, 1865

## Supplementary Material

XML Treatment for Caligus evelynae
